# Development of a rapid and reliable high-performance liquid chromatography method for determination of water-soluble vitamins in veterinary feed premix

**DOI:** 10.14202/vetworld.2021.3084-3090

**Published:** 2021-12-08

**Authors:** Md. Zahangir Hosain, S. M. Shariful Islam, Md. Mostofa Kamal

**Affiliations:** Quality Control Laboratory, Department of Livestock Services, Savar, Dhaka-1343, Bangladesh

**Keywords:** method development, veterinary feed-premix, water-soluble vitamin

## Abstract

**Background and Aim:**

Determination of trace amounts of vitamins in multi-component feed premix is a troublesome analytical procedure. In this study, a simple and rapid high-performance liquid chromatography (HPLC) method was developed and validated for the concurrent detection and quantitation of four water-soluble vitamins such as thiamine, riboflavin, pyridoxine, and cyanocobalamin in veterinary feed premixes.

**Materials and Methods:**

The chromatographic separation of the vitamins was carried out at 35°C temperature on a reversed-phase C18 column using a gradient pump mode. Mobile phase constituents were solvent (a): 25 mM Potassium dihydrogen phosphate and 5 mM sodium hexanesulfonate in deionized water having pH-4.0 and solvent and (b) 5 mM sodium hexanesulfonate in methanol. Detection was performed with HPLC ultraviolet/visible detection set at 278 and 361 nm wavelength in two different channels. The flow rate was 1.2 mL/min and the total run time was 25 min.

**Results:**

The method was validated according to the International Conference on Harmonization and Food and Drug Administration guidelines and acceptance criteria for system suitability, precision, linearity, and recovery were met in all cases. The relative standard deviation for system suitability and precision was <2% for all vitamins. The linearity of the calibration curves was excellent (R_2_>0.999) at concentration of 5, 10, 15, 20, 25, and 30 μg/mL for all vitamins. The limits of detection values were 0.0125, 0.0017, 0.0064, and 0.0065 μg/mL for thiamine, riboflavin, pyridoxine, and cyanocobalamin, respectively, and the limits of quantification values were 0.0378, 0.0051, 0.0213, and 0.0198 μg/mL for thiamine, riboflavin, pyridoxine, and cyanocobalamin, respectively. The recovery percentages ranged from 88% to 115%.

**Conclusion:**

The overall parameters of the proposed method met the validation criteria and this method could be a highly desirable technique for routine analysis of water-soluble vitamins in veterinary feed premix.

## Introduction

Vitamins are critical and biologically active organic compounds that are minor but essential constituents of food required for normal metabolism in human and animal bodies [1,2]. Feed premixes have been widely used in animal nutrition and the manufacture of a feed premix is a significant aspect of veterinary feed production [3]. Even the premixes are designed for the market or used directly for feed production; it is necessary to achieve homogeneity and well balanced of all ingredients. Different types and quantities of micro-ingredients such as vitamins have been used in feed premix production depending on species and categories of animals [4]. Nowadays, contemporary feed production is inconceivable without adding vitamin-mineral premixes. Although, a trace amount of vitamins is required in the diet they are considered essential and their effects on animal performance are vast [5]. There are two groups of vitamins, namely, water-soluble vitamins and fat-soluble vitamins [6]. Water-soluble vitamins such as thiamine, riboflavin, pyridoxine, and cyanocobalamin are involved in many biochemical pathways and help in energy metabolism [7]. However, they only are stored in small amounts and immediately lost with the flow of food or discharged with urine [8]. Therefore, the body needs a continuous supply of water-soluble vitamins to prevent deficiency symptoms and maintain normal body function.

Water-soluble vitamins have been extensively used as vitamin-mineral feed premix in animal production, especially in the poultry industry. The necessity of balanced vitamin-mineral premix in the poultry sector is increasing day by day to optimize the diet compositions and meet the demand of modern poultry production [9]. While vitamin plays a minimal part relative to complete nutrition, they have a key role in the welfare and performance of poultry, along with supporting crucial body functions such as growth performance, reproduction, and immunity [10]. Finished feed is incomplete without supplementation of vitamins and minerals, and it is very important to add a premix in poultry feed to achieve a sufficient level of vitamins and minerals and to prevent the birds from becoming malnourished [11]. They are also used for reducing diseases in animals and poultry [12]. Although the veterinary vitamin-mineral premixes are an important requirement for the rapid and sustainable growth of poultry and livestock production, the question raises about their standard and quality.

However, precise and routine analysis of water-soluble vitamins in veterinary feed premix is challenging due to the complex composition and unstable nature of the target analytes. Many factors can affect the stability of these vitamins such as heat, light, pH, air as well as interactions with other feed components [13,14]. Furthermore, the extraction of these vitamins involves pretreatment through complex chemical reactions, and then a separate method to determine each vitamin. Therefore, the development of a validated analytical method to determine each component of water-soluble vitamins is comparatively a difficult analytical procedure. Some analytical methods such as ultraviolet/visible (UV/Vis) spectrophotometry, fluorimetry, chemiluminescence, capillary electrophoresis, thin-layer chromatography, and liquid chromatography have been offered for the determination of water-soluble vitamins [15,16]. Among these, high-performance liquid chromatography (HPLC) appears promising due to the advancement of both stationary phases and chromatography equipment [17].

The liquid chromatography technique has been widely applied for vitamin analysis from different matrices with some drawbacks such as low reproducibility, longer retention time, and complex mobile phases associated with longer column equilibration, and the total run time of the analysis upper to 60 min [18,19]. Due to the complex composition of the veterinary feed premix and the unstable nature of some vitamins, the extraction and analysis procedure is tedious and time-consuming; sometimes, a special preparation technique is required to obtain the analyte contained in the sample matrix [20]. For this reason, very few standards and validated methods are available for the quantification of water-soluble vitamins in veterinary feed premix.

Hence, the current study aimed to develop a simple, rapid, and validated HPLC technique for the determination of water-soluble vitamins such as thiamine (B_1_), riboflavin (B_2_), pyridoxine (B_6_), and cyanocobalamin (B_12_) in veterinary feed-premix.

## Materials and Methods

### Ethical approval

The present study did not need contact with animals. Hence, ethical approval did not require in this study.

### Study period and location

The study was conducted from December 2019 to July 2021 in the Quality Control Laboratory, Department of Livestock Services (DLS), Dhaka, Bangladesh.

### Chemicals and reagents

Potassium dihydrogen phosphate (KH_2_PO_4_), sodium hexanesulfonate, and methanol (HPLC grade) used in this study were purchased from Sigma-Aldrich and certified reference standard (CRS) of thiamine (Code: Y0000467, Batch: 2.3, ID: 003qpE), riboflavin (Code: R0600000, Batch: 5.0, ID: 007Auc), pyridoxine hydrochloride (Code: P4100000, Batch: 2.0, ID: 001UF1), and cyanocobalamin (Code: C3000000, Batch: 6.0, ID: 00EbaR) were purchased from Europian Pharmacopoeia Reference Standard, Council of Europe, EDQM CS 30026F-67081, Strasbourg, Cedex. Double deionized (DI) water used in this study was obtained from a water deionization plant (ePure-D4642-33, Thermo, USA). All solutions were sonicated and filtered through a 0.45 mm filter using a vacuum filtration unit (Welch, Pall Scientific, USA) before use.

### HPLC method

The high-performance liquid chromatographic system (Waters alliance, Model-e2695XC separation module, 2489 UV/Vis Detector, Waters Corporation, USA) with a data processing unit Empower-3 software was used in this study. HPLC column Waters X-Bridge (4.6×150 mm), 5 mM Packing C18 was used for the separation of vitamins. A gradient pump mode was used where 25 mM KH_2_PO_4_ and 5 mM sodium hexanesulfonate in water having pH-4.0 adjusted with phosphoric acid was used as mobile phase-A and 5 mM sodium hexanesulfonate in methanol was used as mobile phase-B. The flow rate and injection volumes were 1.2 mL/min and 20 μL, respectively, and the column temperature was maintained at 35°C. Wave-length of detection was 278 nm (Channel-A) for thiamine, riboflavin, pyridoxine, and 361 nm (Channel-B) for cyanocobalamin. The whole chromatography was performed at ambient temperature.

### Preparation of standard solution

Weighed accurately 10 mg from each of thiamine, riboflavin, pyridoxine, and cyanocobalamin CRS and transferred into four 100 mL amber color volumetric flasks separately. Initially, 30 mL of DI water were added to each flask and the contents were shaken vigorously by a vortex mixer for 3 min and sonicated for 10 min in an ultrasonic bath. Then, the solutions were diluted to volume with DI water and mixed thoroughly by vortex mixer. These solutions were used as reference stock standard solutions and kept in a refrigerator for further use. Working standard solutions were prepared from stock standard solutions. Before injecting into the liquid chromatography system, the solutions were filtered through a 0.45 mm polyvinylidene difluoride (PVDF) syringe filter.

### Preparation of sample solution

The procedure of standard solution and sample preparation was almost the same. Briefly, 5.0 g of veterinary feed-premix enriched with thiamine, riboflavin, pyridoxine, and cyanocobalamin was accurately weighed and transferred into a 100 mL amber color volumetric flask. Initially, 30 mL of DI water were added to the flask and the contents were shaken vigorously by a vortex mixer for 3 min and sonicated for 10 min in an ultrasonic bath. Then, the content was diluted to volume with DI water and mixed thoroughly by a vortex mixer for 3 min. Thereafter, the mixer was filtered with Whatman 1 Filter paper. Finally, the sample solution was transferred into the sample vial after filtering with a 0.45 mm PVDF syringe filter for liquid chromatography.

### Method validation parameters

Method validation of the present study was performed by measuring the basic parameters of the validation process such as system suitability, precision, linearity, limits of detection (LOD), limits of quantification (LOQ), and recovery. The validation parameters were evaluated using recommended guidelines of the International Conference on Harmonization (ICH) [21] and the United States Food and Drug Administration (FDA) [22,23].

#### System suitability

The system suitability was evaluated by six replicate analyses of a standard aqueous mixture of all vitamins. The acceptance limit of the different parameters for system suitability of the method is calculated according to the guidelines of ICH and FDA where the acceptance criteria are the percentage of relative standard deviation (RSD) for retention time and peak area (PA) is < 2%, the number of theoretical plates (TP) more than 2000, tailing factor (TF), and peak resolution (RS) >2.

#### Precision

The precision of the method was evaluated based on repeatability and intermediate precision. The repeatability was calculated on the results obtained from the same day for six independent mixer solutions of the same concentration (10 μg/mL) and the intermediate precision was evaluated by calculating the repeatability of the same concentration (10 μg/mL) by two analysts on different days. The percentage of RSD was calculated for estimating the precision of this study.

#### Linearity

To evaluate the linearity, six standard mixtures of thiamine, riboflavin, pyridoxine, and cyanocobalamin were prepared and a linear equation was established for each vitamin by plotting the PAs versus the concentrations. Three calibration curves were obtained on three consecutive days with a specified standard concentration of each vitamin. Linearity was calculated by running six standard mixtures of each vitamin, at final concentrations of 5, 10, 15, 20, 25, and 30 μg/mL.

#### LOD and LOQ

The lowest qualitative and quantitative concentrations for the tested linearity range were calculated for each vitamin according to the guidelines of ICH-2000. Both LOD and LOQ were calculated using the expression: k x S.D/b, where k=3.3 for the LOD and 10 for the LOQ, S.D=The standard deviation of the intercept, and b=Slope of the calibration curve tested for linearity.

#### Recovery

The recovery studies were carried out by spiking different amounts (80%, 100%, and 120%) of each vitamin in the pre-analyzed formulation blank matrix sample along with the linearity range. For the estimation of recovery, an accurate amount of each vitamin at three concentration levels (8, 10, and 12 μg/g) was added to approximately 1.0 g of blank matrix powder, and then the powder was extracted and analyzed for recovery using the formula: recovery (%)=(amount obtained/amount spiked)×100.

### Statistical analysis

Data collected in this study were analyzed using Statistical Package for the Social Sciences version 16 statistical package by one-way analysis of variance and by independent samples Student’s t-test. Linear regression analysis was performed using the least square method.

## Results and Discussion

**Figure-1:**
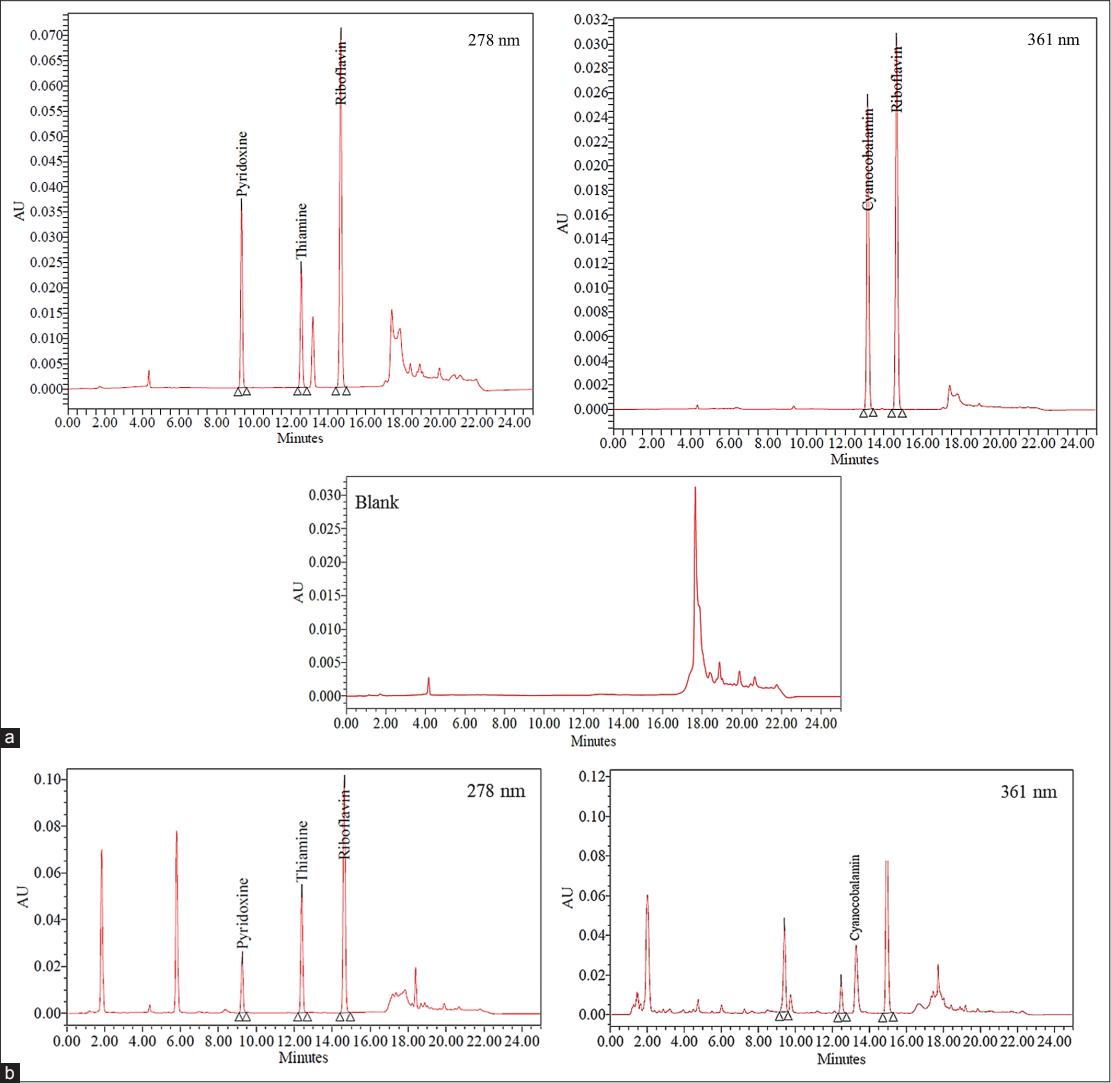
Typical chromatogram of vitamins mix analyzed under standardized conditions. (a) Chromatograms of thiamine, riboflavin, pyridoxine (at 278 nm), and cyanocobalamin (at 361 nm) mixed standard solution; (b) chromatograms of thiamine, riboflavin, pyridoxine (at 278 nm), and cyanocobalamin (at 361 nm) in sample solution of real veterinary feed premix.

This study demonstrates the development and validation of a precise analytical method where the validation criteria [21-23] met in all cases. Representative chromatograms of standard solution and a real feed premix sample are depicted in Figure-1a and b, respectively. The elution of water-soluble vitamins through the analytical column occurs in a specific order and in groups that depend on their chemical properties [24]. As expected, polar vitamins (pyridoxine and thiamine) elute first, followed by low-polar vitamins (cyanocobalamin and riboflavin). The retention time of these four water-soluble vitamins was as follows: 9.34±0.01 min (pyridoxine), 12.55±0.01 min (thiamine), 13.16±0.01 min (cyanocobalamin), and 14.67±0.01 min (riboflavin).

**Figure-2:**
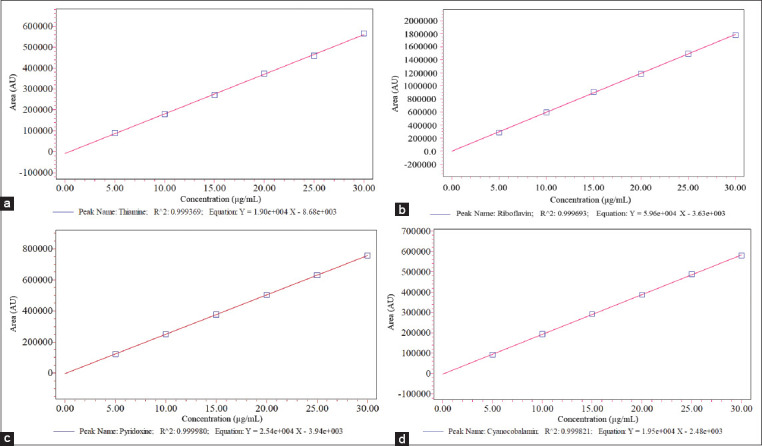
Calibration curve of (a) thiamine, (b) riboflavin, (c) pyridoxine, and (d) cyanocobalamin.

The system suitability parameters (Table-1) reveal that the percentage of RSD for retention time and PA is <2%, the number of TP is more than 2000, and TF and RS are >2, which prove that the values are within the specified limits of the validation process [21-23]. The precision for the method and analyst was evaluated that are shown in Tables-2 and 3. The results reveal that the RSD value for both cases is <2%, which indicates that the proposed method has good reproducibility. From the linearity of Figure-2 and Table-4, it is found that all of the vitamins maintain excellent linearity (R^2^>0.999) within the concentration range of 5-30 μg/mL. The lowest qualitative and quantitative concentrations for the tested linearity range were calculated for each vitamin. The LOD for thiamine, riboflavin, pyridoxine, and cyanocobalamin is found to be 0.0125, 0.0017, 0.0064, and 0.0065 μg/mL, respectively, and the LOQ for thiamine, riboflavin, pyridoxine, and cyanocobalamin is found to be 0.0378, 0.0051, 0.0213, and 0.0198 μg/ mL, respectively, (Table-5). From the results shown in recovery Table-6, it is observed that the percentage recovery values of all the vitamins are between 88% and 115%, which suggests that the method is accurate and it also indicates that the commonly used excipients and additives present in the feed premix formulations are not interfering the proposed method. Many HPLC methods have been published concerning the simultaneous determination of vitamins, but most of the methods are not stable [25-27] which indicates the methods were not properly assessed during stability studies, and some of them are unable to quantify all the water-soluble vitamins simultaneously. Some stable methods were [27,28] reported for the simultaneous quantification of seven water-soluble vitamins in multivitamin syrup preparations but they are unable to analyze cyanocobalamin. Few studies mention the methods of MS-MS detector [27,29], which involves specific and expensive equipment that is not available in all laboratories for routine analysis of these water-soluble vitamins. The method we developed is more precise with good repeatability, intermediate precision, reproducibility, and has high accuracy for detection of all the vitamins used in the study. The sample extraction procedure in our proposed method is simple and quantification of water-soluble vitamins in the real samples is also comparable to the declared values. Thus, the developed method is a simple and reliable approach for simultaneous detection and quantification of four water-soluble vitamins that have a wide range of applications in routine examination of veterinary feed premix.

**Table 1:** System suitability parameters of the proposed high-performance liquid chromatography method.

Vitamin	RT (min)	% RSD of RT	Peak Area	% RSD of Peak Area	Theoretical plates	Tailing factor	Resolution
Thiamine (B_1_)	12.55±01	0.11	175591	0.49	64177	1.04	17.18
Riboflavin (B_2_)	14.67±01	0.01	554275	0.22	77802	1.04	10.43
Pyridoxine (B_6_)	9.34±01	0.15	244745	0.22	43975	1.04	-
Cyanocobalamin (B_12_)	13.16±01	0.09	195841	0.42	6617	1.03	7.28

RT=Retention time, RSD=Relative standard deviation

**Table 2:** Precision under repeatability conditions (n=6).

Vitamin	Retention time % RSD	Area % RSD
Thiamine (B_1_)	0.07	0.83
Riboflavin (B_2_)	0.07	0.47
Pyridoxine (B_6_)	0.08	0.63
Cyanocobalamin (B_12_)	0.08	0.39

RSD=Relative standard deviation

**Table 3:** Intermediate precision (n=6).

Vitamin	First analyst		Second analyst	
	
	Retention time % RSD	Area % RSD	Retention time % RSD	Area % RSD
Thiamine (B_1_)	0.07	0.31	0.07	0.13
Riboflavin (B_2_)	0.05	0.42	0.08	0.09
Pyridoxine (B_6_)	0.09	0.52	0.08	0.15
Cyanocobalamin (B_12_)	0.05	0.68	0.09	0.10

RSD=Relative standard deviation

**Table 4:** Regression parameters of analyzed water-soluble vitamins (regression coefficient, R^2^) in calibration curves.

Vitamin	Regression coefficient		

	1^st^ day	2^nd^ day	3^rd^ day
Thiamine (B_1_)	R^2^=0.9993	R^2^=0.9993	R^2^=0.9993
Riboflavin (B_2_)	R^2^=0.9998	R^2^=0.9996	R^2^=0.9997
Pyridoxine (B_6_)	R^2^=0.9999	R^2^=0.9999	R^2^=0.9999
Cyanocobalamin (B_12_)	R^2^=0.9998	R^2^=0.9998	R^2^=0.9998

**Table 5:** LOD and LOQ values of thiamine, riboflavin, pyridoxine, and cyanocobalamin.

Vitamin	LOD (µg/mL)	LOQ (µg/mL)
Thiamine (B_1_)	0.0125	0.0378
Riboflavin (B_2_)	0.0017	0.0051
Pyridoxine (B_6_)	0.0064	0.0213
Cyanocobalamin (B_12_)	0.0065	0.0198

LOD = Limits of detection, LOQ = Limits of quantification

**Table 6:** Recovery percentages of thiamine, riboflavin, pyridoxine and cyanocobalamin.

Vitamin	Spike % of the vitamin	Spiked conc. (µg/g)	Obtained conc. (µg/g)			Average obtained conc. (µg/g)	Average recovery %

			1^st^ Day	2^nd^ Day	3^rd^ Day		
Thiamine (B_1_)	80	8	6.89	7.72	7.12	7.24	90.53
	100	10	8.48	9.16	8.64	8.76	87.59
	120	12	10.42	11.23	10.55	10.73	89.45
Riboflavin (B_2_)	80	8	6.88	7.63	7.03	7.18	89.78
	100	10	8.79	9.42	8.38	8.86	88.61
	120	12	10.39	11.11	10.32	10.61	88.41
Pyridoxine (B_6_)	80	8	8.44	9.21	9.98	9.21	115.10
	100	10	10.41	9.20	12.07	10.56	105.60
	120	12	13.01	9.22	14.52	12.25	102.08
Cyanocobalamin (B_12_)	80	8	8.45	8.85	7.96	8.42	105.25
	100	10	10.26	10.61	9.87	10.24	102.44
	120	12	12.87	13.42	12.07	12.79	106.55

## Conclusion

In this study, a simple and precise analytical method is developed with an HPLC column for rapid and reliable analysis of water-soluble vitamins in complex veterinary feed premix. The simple technique for sample preparation and mobile phase containing water and methanol make the method more economical and desirable for the quantification of water-soluble vitamins in feed premixes. The recovery of each vitamin with the proposed procedure in the feed premix formulation is also high enough with their respective label claims. Therefore, this method could be a validated analytical procedure for accurate determination of water-soluble vitamins in multi-component veterinary feed premix and might be a precise technique to check the standard and quality of the feed premix intended to be used for increased livestock production.

## Authors’ Contributions

MZH: Conceptualized and designed the study. MZH and SMSI: Generated the data. MZH and SMSI: Analyzed the data. MZH: Drafted the manuscript. MMK: Edited and finalized the manuscript. All authors read and approved the final manuscript.
